# Taurine protection of PC12 cells against endoplasmic reticulum stress induced by oxidative stress 

**DOI:** 10.1186/1423-0127-17-S1-S17

**Published:** 2010-08-24

**Authors:** Chunliu Pan, Grace S Giraldo, Howard Prentice, Jang-Yen Wu

**Affiliations:** 1 Department of Chemistry and Biochemistry, Florida Atlantic University, Boca Raton, FL 33431, USA; 2 Department of Biomedical Science, Florida Atlantic University, Boca Raton, FL 33431, USA

## Abstract

**Background:**

Taurine is a free amino acid present in high concentrations in a variety of organs of mammalians. As an antioxidant, taurine has been found to protect cells against oxidative stress, but the underlying mechanism is still unclear.

**Methods:**

In this report, we present evidence to support the conclusion that taurine exerts a protective function against endoplasmic reticulum (ER) stress induced by H_2_O_2_ in PC 12 cells. Oxidative stress was introduced by exposure of PC 12 cells to 250 uM H_2_O_2_ for 4 hours.

**Results:**

It was found that the cell viability of PC 12 cells decreased with an increase of H_2_O_2_ concentration ranging from approximately 76% cell viability at 100 uM H_2_O_2_ down to 18% at 500 uM H_2_O_2_. At 250 uM H_2_O_2_, cell viability was restored to 80% by taurine at 25 mM. Furthermore, H_2_O_2_ treatment also caused a marked reduction in the expression of Bcl-2 while no significant change of Bax was observed. Treatment with taurine restored the reduced expression of Bcl-2 close to the control level without any obvious effect on Bax. Furthermore, taurine was also found to suppress up-regulation of GRP78, GADD153/CHOP and Bim induced by H_2_O_2_, suggesting that taurine may also exert a protective function against oxidative stress by reducing the ER stress.

**Conclusion:**

In summary, taurine was shown to protect PC12 cells against oxidative stress induced by H_2_O_2_. ER stress was induced by oxidative stress and can be suppressed by taurine.

## Background

Taurine, a sulfur-containing amino acid, is a free amino acid present in high concentrations in a variety of organs of most mammals, including brain, heart, kidneys [1]. Taurine mediates many physiological functions, such as neuro-modulation, regulation of calcium-dependent processes, osmoregulation, thermoregulation, membrane stabilization and detoxication, neurotransmission and neuroprotection [2-6]. Taurine is known as an antioxidant to counteract oxidative stress, which is involved in many diseases, such as chronic lung disease, diabetes, Alzheimer's disease, Parkinson's disease and heart failure [7,8]. A recent paper revealed that taurine plays an important role in reducing ER stress in C2C12 and 3T3L1 cells [9].

The ER is a key cell organelle that is responsible for synthesis and folding of proteins destined for secretion, cell membrane, Golgi apparatus, lysosomes and elsewhere, intracellular calcium homeostasis, and cell death signaling activation [10]. Physiological or pathological processes that disturb protein folding in the ER lumen are referred to as ER stress, and a set of signaling pathways responding to ER stress is termed the Unfolded Protein Response (UPR) [11]. ER stress has been recently implicated in inflammation, ischemia, heart disease, liver disease, kidney disease and neurodegenerative diseases, which include Parkinson’s, Alzheimer’s disease and polyglutamine disease [12-14]. The predominant signaling pathways associated with ER stress are initiated by the ER membrane-associated proteins, protein kinase R [PKR]-like ER kinase (PERK), inositol requiring enzyme 1 (IRE1), and activating transcription factor 6 (ATF6), which in turn activate distinct signaling cascades mediating the ER stress response [15-17]. Among these three major UPR signal transduction pathways, the IRE-1 and ATF-6 pathways increase the expression of the ER-resident chaperone, glucose-regulated protein 78 (GRP78) [18,19], and all of these three pathways up-regulate the transcription factor C/EBP homologous protein (CHOP), also known as growth arrest and DNA damage-inducible gene 153 (GADD153) [20]. CHOP/GADD153 regulates expression of several Bcl-2 family members. For example, CHOP decreases expression of antiapoptotic Bcl-2 [21], but increases expression of the proapoptotic Bim [22], thus contributing to cell death. The PERK pathway can also activate caspase-12, which plays an essential role in programmed cell death progression during the proapoptotic phase of the ER stress response [23]. Recently, it has been suggested that oxidative stress and ER stress are closely linked events, although the molecular pathways that couple these processes are poorly understood [24]. Moreover, GRP78 was shown to protect neurons against excitotoxicity and to suppress oxidative stress [25]. In the present study, we demonstrated that taurine exerts a protective function against ER stress induced by oxidative stress in PC 12 cells.

## Methods

### Materials

F-12K media, trypsin-EDTA solution, horse serum and rat phenocromocytoma PC12 cell line were purchased from ATCC (Manassas, VA, USA). Fetal bovine serum, poly-D-lysine, taurine, Penicillin-Streptomycin and other chemicals were purchased from Sigma (St. Louis, MO, USA). Mouse anti-actin, rabbit anti-Bax, rabbit anti–Bcl-2, rabbit anti-GRP78, rabbit anti-CHOP/GADD153 antibodies, and secondary mouse and rabbit antibodies were purchased from Santa Cruz Biotechnology (Santa Cruz, CA, USA). Rabbit anti-Bim antibody was purchased from assay designs (Ann Arbor, Michigan, USA). Adenosine 5’-triphosphate (ATP) Bioluminescent Assay Kit and 3, (4, 5-dimethylthiazol-2-yl) 2, 5-diphenyl-tetrazolium bromide (MTT) assay kit were purchased from Promega (Madison, WI, USA) and ATCC (Manassas, VA, USA) respectively. RIPA buffer was purchased from thermo scientific (Rockford, IL, USA).

### Cell culture

PC12 cells were maintained at 37^o^C/5% CO_2_ in F12-K medium supplemented with 2.5% (v/v) fetal bovine serum (FBS), 15% (v/v) heat-inactivated horse serum (HS) and 1% (v/v) penicillin-streptomycin solution. All experiments were performed on undifferentiated cells plated in 96-well plates at a density of approximately 5×10^4^ cells/ml for the ATP assay, 1×10^5^ cells/ml for the MTT assay and in 60mm petri dishes at 5×10^5^ cells/well for western blot for 24 hours before starting the experiments. The 96-well plates or petri dishes were precoated with poly-D lysine before plating.

### Measurement of cell viability

#### ATP assay

PC12 cells in 96-well plates were treated with or without 25 mM taurine for 1 hour, and then cells were exposed to 100-500 uM H_2_O_2_ for 4 hours to induce cell death. ATP solution (Promega) was added to each well and cells were incubated for 10 minutes, then the amount of ATP was quantified through a luciferase reaction. The luminescence intensity was determined using a luminometer (SpectraMax, Molecular Devices) after transferring the lysate to a standard opaque walled multi-well plate. The ATP content was determined by running an internal standard and expressed as a percentage of untreated cells (control).

#### MTT assay

PC12 cells in 96 well plates were treated with 25 mM Taurine for 1 hour and then cells were exposed to 250 uM H_2_O_2_ for 4 hours to induce cell death. Subsequently, 10 ul MTT reagent (ATCC) was added to each well and cells were incubated for 4 hours until a purple precipitate was visible. Then 100 ul detergent reagent was added and the solution was left at room temperature in the dark for 2 hours. The absorbance was detected with a microtiter plate reader at 570 nm.

### Western blot analysis

PC12 cells were lysed in RIPA buffer (25 mM Tris_HCl pH 7.6, 150 mM NaCl, 1% NP-40, 1% sodium deoxycholate, 0.1% SDS) containing 1% (v/v) mammalian protease inhibitor cocktail from Sigma and separated on SDS-PAGE, following by transferring to a nitrocellulose membrane. The membrane was then blocked in blocking buffer (20 mM Tris-HCl, 150 mM NaCl, 0.1% Tween-20, 5% milk) for 1.5 hours at room temperature. After blocking, corresponding primary antibody was incubated for one hour, followed by a one hour incubation with the corresponding HRP-conjugated secondary antibody at room temperature. Extensive washes with a blocking buffer were performed between each step. The protein immuno-complex was visualized by ECL detection reagents.

### Statistical analysis

All data presented in the figures were expressed as the mean±SEM. The Student’s t-test or one-way ANOVA was used to compare means between groups. Differences of P<0.05 were considered statistically significant.

## Results

### Dose dependent toxicity of H_2_O_2_ in PC12 cells

**Figure 1:**
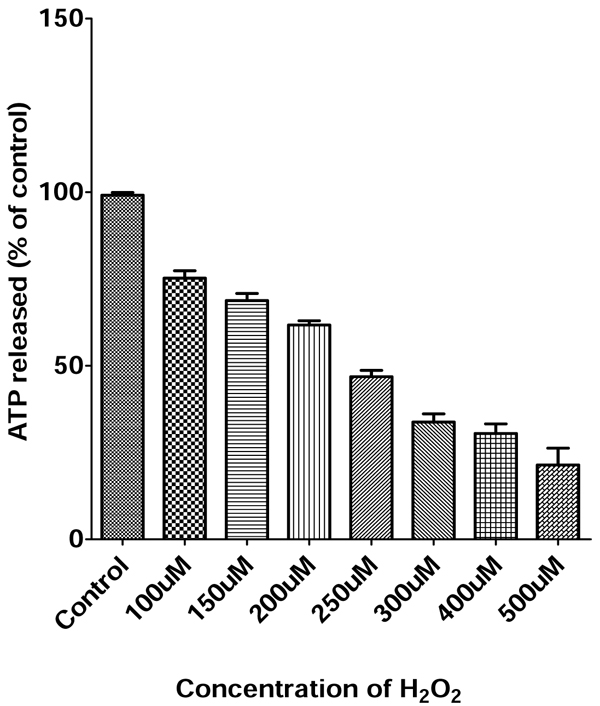
**Dose-dependence of H_2_O_2_-induced cell injury in PC12 cells measured by ATP assay.** PC12 cells were exposed to H_2_O_2_ ranging from 0 - 500 uM for 4 hours, followed by ATP assay to measure the extent of cell viability.

The PC12 cells were exposed to different concentrations of H_2_O_2_ in a range of 100-500 uM for 4 hours, then the ATP assay was performed. As expected, the survival of PC12 cells decreased with the increasing of concentrations of H_2_O_2_ from 76% at 100 uM H_2_O_2_ to 18% at 500 uM H_2_O_2_ as demonstrated in Fig. 1. We chose the optimal dose of 250 uM H_2_O_2_ for cell viability test. At 250 uM H_2_O_2_, approximately 45% of the PC12 cells remained viable.

### Taurine protected PC12 cells against H_2_O_2_ induced oxidative stress

**Figure 2:**
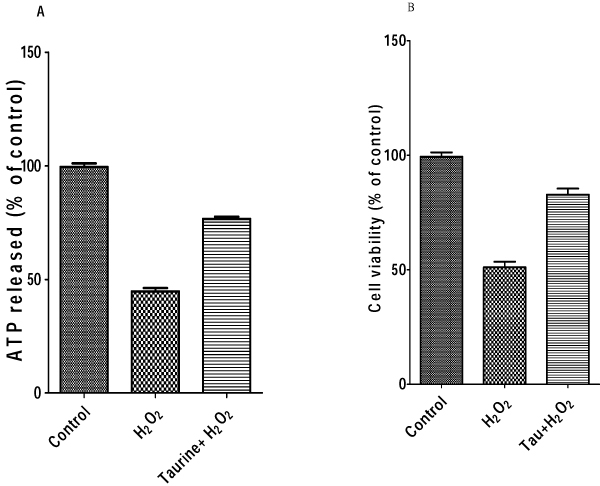
**Effect of taurine on H_2_O_2_ induced cell injury in PC12 cells.** A, Cell viability was measured by ATP assay; B, Cell viability was measured by MTT assay. 25 mM taurine was preincubated for 1 hour followed by 250 uM H_2_O_2_ treatment for 4 hours.

In our previous paper, it was revealed that preincubation with 25 mM taurine resulted in maximal recovery from neuronal injury induced by glutamate [5]. For this reason, we chose 25 mM as the optimal concentration of taurine. To determine the protective effect of 25 mM taurine on 250 uM H_2_O_2_ induced oxidative stress, cell viability was examined using the ATP assay and MTT assay, respectively, as shown in Fig. 2 (A and B). Lane 2 in both Fig. 2A and 2B showed that 250 uM H_2_O_2_ significantly decreased the survival of PC12 cells to about 45-50%. The cell viability is up to 75-80%, as shown in Fig. 2 (lane 2 in 2A, lane 2 in 2B) after treatment with 25 mM taurine for 1 hour, following exposure to 250 uM H_2_O_2_ for 4 hours.

### Extracellular taurine elicited protection of PC12 cells against H_2_O_2_ induced oxidative stress

**Figure 3:**
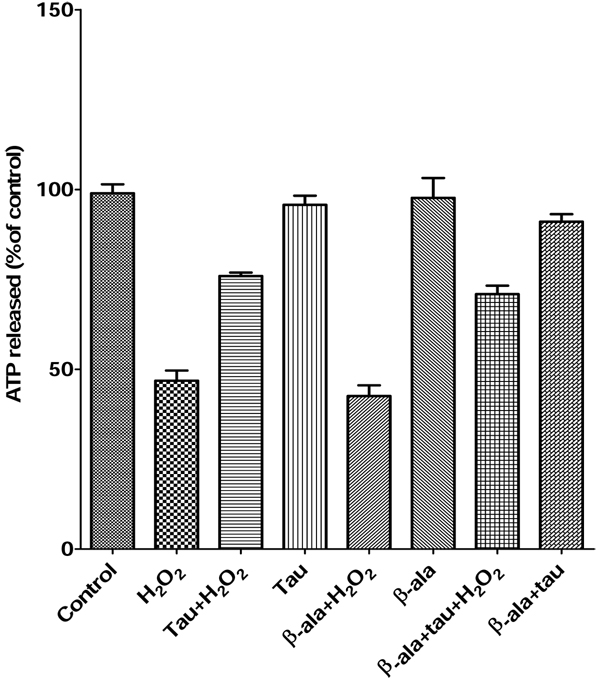
**Effects of β-alanine (β-ala), an analog of taurine (tau) on H_2_O_2_-induced cell injury in PC12 cells measured by ATP assay.** PC12 cells were preincubated with 25 mM taurine, 25 mM β-alanine or combination of 25 mM taurine and 25 mM β-alanine for 1 hour followed by treatment with or without 250 uM H_2_O_2_. The extent of cell viability was measured by ATP assay.

Taurine uptake across the cell membrane has been found to be dependent on Na^+^/Cl^-^ dependent TauT or H^+^ - coupled PAT1 transporters [26,27]. In order to investigate whether intracellular or extracellular taurine has a protective effect, β- alanine, the analogue of taurine, was utilized as a competitive inhibitor of the taurine transporters [28]. The PC12 cell viability was tested by ATP assay, as shown in Fig. 3. To compare the PC12 cell viabilities from treatment with taurine and β- alanine, we applied 25 mM β- alanine, which was at the same concentration as taurine. Cell survival was similar with or without β- alanine, indicating that β- alanine afforded no protective effect against the toxicity of H_2_O_2_ (Fig. 3, lane 2 and lane 5).The ATP released with the 25 mM taurine preincubation after exposure to H_2_O_2_ was similar to that of 25 mM taurine and 25 mM β- alanine, which indicated that extracellular taurine protected the PC12 cells against oxidative stress induced by H_2_O_2_ (Fig. 3, lane 3 and lane 7). Results from PC12 cell survival measurement showed no significant differences between conditions with or without 25 mM taurine or 25 mM β- alanine or taurine + β- alanine, indicating that 25 mM β- alanine has no effect on the PC12 cells, as determined by the ATP assay (Fig. 3 lane 4, 6 and 8).

### Taurine restored the expression of Bcl-2 and had no significant effect on the expression of Bax

**Figure 4:**
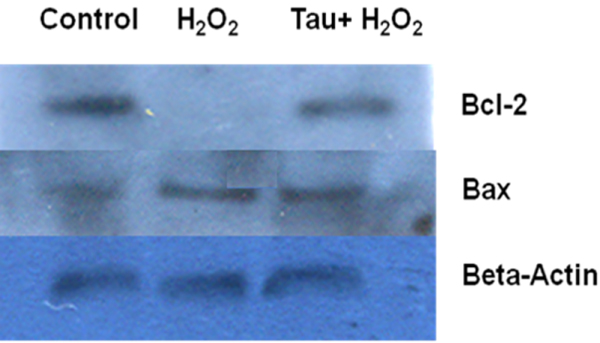
**Effect of taurine on the expression of Bcl-2 and Bax in H_2_O_2_-treated PC12 cells.** PC12 cells were treated with or without 25 mM taurine before treatment with or without 250 uM H_2_O_2_ for 4 hours followed by Western blot analysis with anti-Bcl-2 or anti-Bax antibodies. Beta-actin was included to show equal loading.

Bcl-2 and Bax are two proteins which belong to the Bcl-2 family that modulate cell survival. It has been demonstrated that cell survival is modulated at least in part by the Bcl-2 family of proteins: apoptosis-inhibiting gene products, Bcl-2 and Bcl-xL and apoptosis-accelerating gene products, Bax and Bad [29,30]. The level of Bcl-2 was decreased, but Bax was only slightly increased in PC12 cells under treatment with 250 uM H_2_O_2_ for 4 hours (Fig. 4, lane 2). In summary, the Bcl-2 level was restored after preincubation with taurine, but there was no significant change in the Bax level (Fig. 4, lane 3).

### Taurine reversed the H_2_O_2_-induced up-regulation of GRP78, CHOP/GADD 153 and Bim in PC12 cells

**Figure 5:**
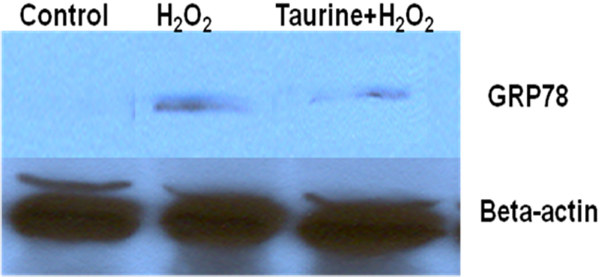
**Effect of taurine on the expression of ER stress marker GRP78 in H_2_O_2_-treated PC12 cells.** PC12 cells were treated with or without 25 mM taurine before treatment with or without 250 uM H_2_O_2_ for 4 hours followed by Western blot analysis with anti-GRP78 antibodies. Beta-actin was included to show equal loading.

**Figure 6:**
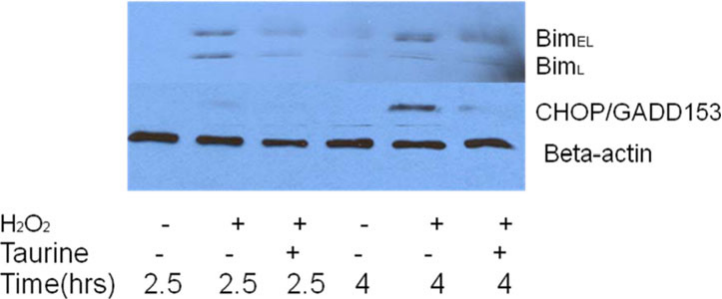
**Effect of taurine on the expression of Bim and CHOP/GADD153 in H_2_O_2_-treated PC12 cells.** PC12 cells were treated with or without 25 mM taurine before treatment with or without 250 uM H_2_O_2_ for 4 hours followed by Western blot analysis with anti- Bim or anti-CHOP/GADD153 antibodies. Beta-actin was included to show equal loading.

Western blot analysis showed that the UPR regulator GRP78 was up-regulated after treatment with H_2_O_2_ for 4 hours, indicating that ER stress was induced by oxidative stress (Fig. 5 lane 2). Taurine suppressed the expression of GRP78 protein in PC12 cells after exposure to H_2_O_2_ (Fig. 5 lane 3). To determine if oxidative stress-induced apoptosis was also activated by the ER stress pathway, we examined expression of the ER stress apoptotic factor, CHOP. CHOP expression is minimal and barely detectable under normal homeostatic conditions [30], and upregulation of CHOP has been reported to signal the activation of ER stress-mediated apoptosis [20,21,31]. Here, we characterized the processing of CHOP/GADD153 in PC12 cells by Western blotting of whole cell lysates after treatment with H_2_O_2_ for 2.5 and 4 hours, as shown in Fig. 6. Western blot analyses revealed a minimal level of CHOP expression at 2.5 hours exposure to H_2_O_2_, and pronounced CHOP expression at 4 hours of treatment by H_2_O_2_ alone. There was a remarkable reduction in CHOP protein expression in PC12 cells treated with taurine, following by H_2_O_2_ treatment, compared to cells exposed to H_2_O_2_ only (Fig. 6). Recently, ER stress was shown to up-regulate Bim protein level through CHOP/GADD153 mediated direct transcriptional induction [22]. Our results demonstrated that two longer isoforms of Bim, Bim_EL_ and Bim_L_ in PC12 cells treated with H_2_O_2_ were highly expressed compared to controls without any treatment. Taurine suppressed the expression of Bim_EL_ and Bim_L_ in PC12 cells treated with H_2_O_2_ for 2.5 hours and 4 hours, as shown in Fig. 6.

## Discussion

This paper presents evidence indicating that 1) extracellular taurine exerted a protective function against oxidative stress induced by H_2_O_2_ in PC12 cells, 2) Bcl-2 expression in PC12 cells was restored but not Bax expression after treatment with taurine, 3) H_2_O_2_ induced ER stress by up-regulation of GRP78, Bim and CHOP/GADD153 and 4) Taurine protected PC12 cells from ER stress induced by H_2_O_2_ through downregulation of GRP78, Bim and CHOP/GADD153.

As a naturally occurring antioxidant, taurine was investigated to treat oxidative stress trigged by different inducers, such as age-related retinal degeneration, a high cholesterol diet, lead poisoning and nitric oxide [32-35]. Although a number of studies proved that taurine has a protective function against oxidative stress, the mechanism underlying its protection is still not fully understood. Li et al. (2009) [36] demonstrated that taurine treatment alleviated the oxidative injury of the kidney, improved SOD and GSH-Px activities and prevented mitochondrial membrane injury. They found that taurine protected the kidney from oxidative injury through a mitochondrial-linked pathway [36]. Here, we demonstrate that the function of taurine as a protectant against oxidative stress induced by H_2_O_2_ is via the alleviation of ER stress.

**Figure 7:**
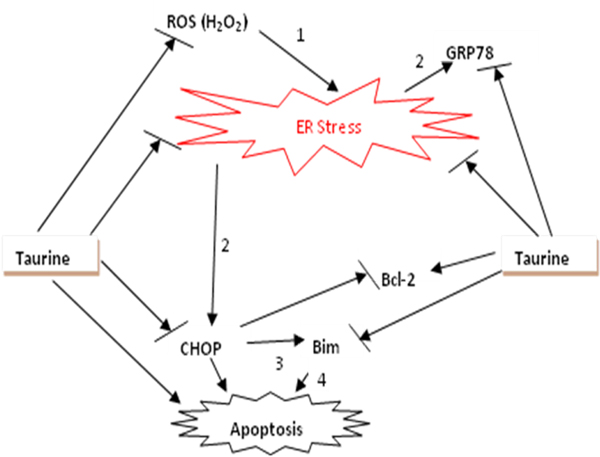
**Proposed mechanism of taurine protection against ER stress induced by oxidative stress in PC12 cells.** There are four stages to the process. 1, ROS (H_2_O_2_) induces ER stress; 2, this leads to up-regulation of the ER stress marker GRP78 and CHOP/GADD153. 3, CHOP induces apoptosis and up-regulation of BH3-only proapoptotic protein Bim and inhibits the expression of Bcl-2. 4, Bim induces cell apoptosis. Taurine exerts its protection against oxidative stress induced by ROS (H_2_O_2_). Taurine suppresses ER stress induced by oxidative stress through down-regulating the expression of GRP78 and CHOP. Furthermore, taurine protects PC12 cells by up-regulation of Bcl-2 and down-regulation of Bim.

It has been confirmed that the overproduction of reactive oxygen species (ROS), such as superoxide (O_2_^-^) and hydrogen peroxide (H_2_O_2_), contributes to damage of lipids, proteins, carbohydrates and nucleotides [37]. ER, acting as a protein folding compartment, is susceptible to oxidative stress [38]. In recent years, people noticed that there is a functional link between oxidative stress and ER stress [39,40]. Hayashi et al. (2005) found that oxidative ER damage is implicated in ischemic neuronal cell death by investigating changes in activating transcription factor-4 (ATF-4) and CHOP expression [38]. He *et al* (2008) [41] showed that the oxidant tert-butyl hydroperoxide increases oxidative stress, increases accumulation of ROS in the ER, and upregulates expression of GRP-78 and GADD153 in human retinal pigment epithelium cells. These studies provide further evidence of the connection between oxidative stress and ER stress. Interestingly, it was found that ER stress protected renal epithelial cells against oxidative stress by preventing the increase in intracellular Ca^2+^concentration that normally follows H_2_O_2_ exposure [42]. Zhang (2010) [39] proposed that ROS can target ER-based calcium channels and chaperones, leading to the release of calcium from the ER to the cytosol. Increased cytosolic calcium can stimulate mitochondrial metabolism to produce more ROS. Mitochondrial ROS can further accentuate calcium release from the ER, which leads to the further accumulation of toxic levels of ROS. At the same time, perturbation of ER calcium homeostasis can disrupt the protein folding process, inducing ER stress and the activation of the UPR [40]. In the present study, we give supportive evidence that oxidative stress induced by H_2_O_2_ contributes to ER stress. Based on the previous findings and the current results, we propose a model for the mechanism of taurine protection against ER stress induced by oxidative stress. This model is presented in Fig. 7.

## Conclusion

In summary, the results of our present study shed a light on the sequential relationship between oxidative stress and ER stress and the mechanism underlying protection by taurine against oxidative stress. Further study is warranted to examine in detail which specific pathway in ER can be activated or inhibited by taurine. In addition, it remains to be known exactly how oxidative stress triggers ER stress. Further scrutiny is necessary to elucidate the precise mechanism behind the oxidative stress-elicited ER stress. Our model suggests a key role for extracellular taurine in preventing ER stress induced by H_2_O_2_.

## Competing interests

The authors declare that they have no competing interests.
